# Integrative RNA-seq and CLIP-seq analysis reveals *hnRNP-F* regulation of *TNFα/NFκB* signaling in high-glucose conditions

**DOI:** 10.3389/fphys.2025.1475441

**Published:** 2025-09-09

**Authors:** Lan Wang, Huimeng Li, Xinyuan Guo, Xiaoqin Wang

**Affiliations:** ^1^ Hubei University of Chinese Medicine, Wuhan, China; ^2^ Hubei Provincial Hospital of Traditional Chinese Medicine, Wuhan, China; ^3^ Affiliated Hospital of Hubei University of Chinese Medicine, Hubei Key Laboratory of Theory and Application Research of Liver and Kidney in Traditional Chinese Medicine, Wuhan, China; ^4^ Hubei Shizhen Laboratory, Wuhan, China

**Keywords:** *hnRNP-F*, diabetic kidney disease, RNA-seq, differential gene expression, variable splicing, *TNFα-NFκB* signaling pathway

## Abstract

**Background:**

Diabetic kidney disease (DKD), with its complex pathogenesis, is the most important cause of end-stage renal disease and has become an urgent public health problem worldwide. Heterogeneous nuclear ribonucleoprotein F (*hnRNP-F*) is a member of a subfamily of widely expressed nuclear heterogeneous ribonucleoproteins with biological roles in regulating gene expression and variable splicing. Some studies have investigated *hnRNP-F* in DKD. However, its potential mechanism in renal intrinsic cells has rarely been reported. Therefore, it is necessary to further investigate its potential mechanism in DKD in the search for novel ideas for new therapeutic targets for DKD.

**Methods:**

In this study, *hnRNP-F* was overexpressed in human renal proximal tubular epithelial (HK-2) cells cultured in high-glucose conditions, while an empty vector was transfected into HK-2 cells as a control group (NC). Meanwhile, to avoid any osmotic stress that might be caused by the use of high sugar, we also added mannose as a non-osmotic control. RNA-seq was utilized to generate transcriptome data following *hnRNP-F* overexpression, allowing for the analysis of differential gene expression and alternative splicing events influenced by *hnRNP-F* overexpression. Similarly, we overexpressed *hnRNP-F* in mouse podocyte clone 5 (MPC5) cells and verified the relevant indicators using Western blotting (WB) under high-glucose and high-mannitol conditions, respectively. We also downloaded the CLIP-seq data of *hnRNP-F* in human 293T cells from the Gene Expression Omnibus (GEO) database. Through integrative analysis of RNA-seq and CLIP-seq, we tried to identify a set of potential direct targets of *hnRNP-F* in cells.

**Results:**

In this study, RNA sequencing (RNA-seq) was utilized to demonstrate that the upregulation of *hnRNP-F* in HK-2 cells cultured under high-glucose conditions resulted in a substantial decrease in the expression of genes associated with the inflammatory response and suppression of the *TNFα-NFκB* signaling pathway. This was also verified in MPC5 cells. By analyzing CLIP-seq and RNA-seq data, we found that *hnRNP-F* may inhibit gene expression by binding to lncRNA *SNHG1*. Conversely, this upregulation led to a significant increase in alternative splicing events of genes implicated in DKD, such as *hnRNPA2B1, OSML*, *UGT2B7*, *TRIP6*, and *IRF3*. Combining CLIP-seq data, we found that *hnRNP-F* binds to and regulates variable splicing of the *hnRNP* protein family and splicing factors. This result suggests that *hnRNP-F* may regulate alternative splicing through the coordinated action of multiple splicing factors.

**Conclusion:**

*hnRNP-F* has dual functions in mRNA transcriptional and post-transcriptional levels and may bind with lncRNA *SNHG1* to negatively regulate the transcription of genes involved in the *TNFα/NFκB* signaling pathway. Meanwhile, *hnRNP-F* may function in the co-regulation of alternative splicing events in cells by interacting with ZFP36 to form a complex.

## 1 Introduction

Diabetic kidney disease (DKD) is a global public health problem and an important cause of chronic kidney disease (CKD), leading to end-stage renal disease, and urgently needs our in-depth research and effective response (Chen et al., 2020; Barrera-Chimal and Jaisser, 2020). Various factors are implicated in DKD progression, including autoimmunity, inflammation, renal fibrosis, renal hemodynamic alterations, mitochondrial dysfunction, abnormalities in glucolipid metabolism, oxidative stress, and epigenetic inheritance (Tuttle et al., 2022; Lin et al., 2018). Therefore, drugs targeting inflammatory and fibrotic pathways have important therapeutic implications in DKD research (Lytvyn et al., 2020).

Heterogeneous nuclear ribonucleoprotein F (*hnRNP-F*) is a subfamily of widely expressed *hnRNPs*. The proteins of this subfamily are RNA-binding proteins (RBPs) that interact with heterogeneous nuclear RNAs. In addition, as splicing factors, *hnRNPs* are involved in various aspects of RNA metabolism, including alternative splicing of target RNAs, polyadenylation, sequence editing, RNA transport, RNA stabilization and degradation, intracellular localization, and translational control (Smith et al., 2021; Huang et al., 2017; Ladd, 2016). Alternative splicing (AS) is a major mechanism for generating multiple structurally and functionally different proteins from a single gene, greatly expanding proteome diversity (Cheng et al., 2021). In humans, approximately 95% of multiexon genes undergo AS, and a recent study demonstrated that splice isoform switching is critical in the various kidney diseases, especially in DKD (Zhou et al., 2024; Liao et al., 2024).

Based on the biological role of *hnRNP-F* in regulating gene expression and AS, its role in DKD has gradually received attention. At present, scholars have confirmed the closer link between *hnRNP-F* and DKD to varying degrees in various experiments. For example, in patients with type 2 diabetes, the protein levels of *hnRNP-F* have significantly decreased in renal cortex tissues. It shows that *hnRNP-F* is involved in mediating insulin inhibition of Bcl2 modifier expression and diabetic tubulopathy (Ghosh et al., 2019). *hnRNP-F* protects the kidney from oxidative stress and nephropathy by stimulating Sirtuin-1 expression and signaling in diabetic mice (Ghosh et al., 2019). Overexpression of *hnRNP-F* attenuates *TGF-β1*-induced diabetic kidney injury in mice, mainly by stimulating renal Ace-2 gene expression (Lo et al., 2015). *hnRNP-F* was recently found to have a protective effect against podocyte injury, and *hnRNP-F* deficiency promotes podocyte pathology through activation of Mettl14 expression and inhibition of Sirt1 expression by its nuclear translocation (Liao et al., 2024).

In the study of DKD, HK-2 cells, as a model of human proximal tubular epithelial cells, are widely used to investigate the mechanisms of diabetes-induced tubular damage and potential therapeutic strategies (Darshi et al., 2024). In the present study, renal tubular epithelial cells (HK2) overexpressing *hnRNP-F* were cultured in high-glucose conditions, while a control group (NC) was similarly exposed to high-glucose. Mannitol was added to the media as an osmotic control. Subsequently, transcriptome data were acquired through RNA sequencing (RNA-seq) following the overexpression of *hnRNP-F* under high-glucose conditions. The expression of differentially expressed genes linked to inflammation was confirmed in both db/db and db/m mouse models. Additionally, overexpression of *hnRNP-F* in conditionally immortalized mouse podocyte cell line (Clone 5) (MPC5) confirmed its inhibitory effect on the *TNF-α/NF-κB* inflammatory signaling pathway. This approach enabled the analysis of differential gene expression and AS events influenced by the overexpression of *hnRNP-F*. Furthermore, the anti-inflammatory effect of *hnRNP-F* has been experimentally demonstrated under LPS stimulation. The results show that *hnRNP-F* broadly regulates gene expression and alternative splicing related to diabetic nephropathy, particularly in inflammation-related pathways, offering new insights into DKD gene regulation.

## 2 Materials and methods

### 2.1 Cell lines and cell culture

HK-2 cells (Cell Bank of China Academy of Sciences) were cultured in DMEM/F12 (Gibco) supplemented with 10% FBS (Gibco) and 1% penicillin/streptomycin (MilliporeSigma). MPC-5 cells (Procell) were maintained in RPMI 1640 (Procell) containing 10% FBS (Gibco) and 1% penicillin/streptomycin (MilliporeSigma). Both cell lines were incubated at 37 °C under 5% CO_2_. Upon reaching 50% confluency, cells were treated with 30 mM high-glucose (HG; MilliporeSigma) for 72 h. Mannitol (MilliporeSigma) served as an osmotic control. LPS (Solarbio) was dissolved in sterile PBS to prepare a 10-mg/mL stock solution. HK-2 cells (10 μg/mL) and MPC5 cells (45 μg/mL) were treated with these LPS solutions for 24 h to model cell injury. Cells were subsequently harvested for Western blot analysis of target protein expression.

### 2.2 *hnRNP-F* was overexpressed in HK-2 and MPC5 cell lines

We employed four parallel wells for each group of HK-2 cells: HK-2 cells transfected with the control lentivirus were cultured in a high-glucose medium containing 30 mM glucose (HG-NC) for 72 h, while a separate group of control lentivirus-transfected HK-2 cells was cultured in a medium containing 30 mM mannitol to serve as an osmotic control (OS-NC). Similarly, HK-2 cells transfected to overexpress the *hnRNP-F* lentivirus (Gene ID:98758, Lentiviral expression vector LV5) were maintained in a 30 mM glucose medium (HG-OE) and in a mannitol medium (OS-OE) for 72 h, respectively. MPC5 cells were cultured under HG conditions (30 mM, 72 h), mannitol treatment (30 mM, 72 h), or LPS stimulation (10 μg/mL, 24 h) and then transfected with an *hnRNP-F* overexpressing plasmid packaged in a lentiviral vector (Gene ID: 98758, vector name: HBLV-ZsGreen-PURO).

### 2.3 Reverse transcription quantitative real-time PCR (RT-qPCR)

Total RNA was isolated from the renal cortex and cells separately using the TRIzol method, and 1 μL of total RNA was used as the template, reverse-transcribed to cDNA, and continued to be amplified by using cDNA as the template, sequentially, at 95 °C for 3 min, 1 cycle, 95 °C for 10 s, and 62 °C for 40 s, for a total of 40 cycles. The mRNA levels of HG-NC and HG-OE were determined using the 2^−ΔΔCT^ method, with *β-actin* and *GAPDH* serving as internal references. Similarly, mRNA levels in renal tissues of *db/db* and *db/m* mice were calculated. The specific primer sequences are shown in Table 1.

**TABLE 1:** Primer sequences for qRT-PCR.

Gene	Forward primer (5′–3′)	Reverse primer (5′–3′)
HOMO-*hnRNP-F*	CTCCGTCGTGGAAGCAGG	CGAGCAGGACTGGTTTCTGT
HOMO-*GAPDH*	TCGGAGTCAACGGATTTGGT	TTCCCGTTCTCAGCCTTGAC
HOMO*-β-actin*	CACCCAGCACAATGAAGATCAAGAT	CCAGTTTTTAAATCCTGAGTCAAGC
Mouse-*hnRNP-F*	GCCTTCGTTCAGTTTGCCTC	AATGCCAATGTACCTCCGGG
Mouse-*GAPDH*	AACGACCCCTTCATTGAC	GAAGACACCAGTAGACTCCAC
Mouse-*β-actin*	TGTACCCAGGCATTGCTGAC	AACGCAGCTCAGTAACAGTCC
Mouse-*GDF15*	GCAGACTTATGATGACCTGGTGG	AAGGGGAGTGTAGGTGAGGAGC
Mouse*-IL6*	CCCCAATTTCCAATGCTCTCC	CGCACTAGGTTTGCCGAGTA
Mouse*-PTX3*	CTCAGTTCCCAGTCCCTAGTGTTG	GGAGTCCACCCTCAGGAACAGA
Mouse*-TFPI2*	CTCCAGTCCAAAGGATGAAGGT	AGTTATTCTCATTCCCACCACAGC
Mouse-*GAPDH*	CCTCGTCCCGTAGACAAAATG	TGAGGTCAATGAAGGGGTCGT
Mouse*-β-actin*	TGGTCTTTCTGGTGCTTGTCTC	CAGTTCAGTATGTTCGGCTTCC

### 2.4 Co-immunoprecipitation (Co-IP)

After extracting the proteins from the cells OE-*hnRNP-F* and the NC HK-2 cells, the lysates were pre-cleared with rabbit IgG (3 μg/mg protein) and protein A/G magnetic beads. Then, they were incubated overnight at 4 °C with anti-*hnRNP-F* or control IgG (3 μg/mg protein). The complexes were captured with fresh magnetic beads (20 μL/500 μL lysate, room temperature for 2 h), washed three times with lysis buffer, and eluted in 1× Laemmli buffer (95 °C, 5 min) for immunoblotting.

### 2.5 Western blotting analysis

Total protein lysates (30 µg/lane) from cells or renal tissues were separated by 10% SDS-PAGE and transferred to PVDF membranes (Millipore). After blocking with 5% non-fat milk/TBST for 1 hour, the membranes were incubated overnight at 4°C with the following primary antibodies:*hnRNP-F*, *β-actin*, *GAPDH* (1:5000; Proteintech, 67701-1-Ig, 20536-1-AP, 60004-1-Ig), *p-p65* (1:500; Invitrogen, MA5-15160), *p65* (1:5000; Abclonal, A19653) and *TNF-α* (1:1000; Abcam, ab183218). Following TBST washes (3 × 10 min), membranes were incubated 1 h with HRP-conjugated secondary antibodies: Goat anti-mouse, Goat anti-rabbit (1:1000; Proteintech, SA00001-1, SA00001-2). Signals were detected by ECL (Proteintech, P0018S) and quantified using ImageJ (NIH, v1.53e), normalized to *β-actin*/*GAPDH*.

### 2.6 RNA extraction and sequencing

All RNA was processed with RQ1 DNase (Promega) to remove DNA. The quality and quantity of the purified RNA were determined by measuring the absorbance at 260 nm/280 nm (A260/A280) utilizing SmartSpec Plus (BioRad). RNA integrity was further verified by 1.5% agarose gel electrophoresis.

For each sample, 1 μg of total RNA was used for RNA-seq library preparation. mRNAs were captured by VAHTS mRNA capture beads (Vazyme, N401). The purified RNA was treated with RQ1 DNase (Promega) to remove DNA before being used for directional VAHTS with a Universal V8 RNA-seq Library Prep Kit for Illumina (NR605). Polyadenylated mRNAs were purified and fragmented. Fragmented mRNAs were converted into double-stranded cDNA. Following end repair and A tailing, the DNAs were ligated to Adapter (N323). After purification of the ligation product and size fractioning to 300–500 bps, the ligated products were amplified and purified, then quantified and stored at −80 °C before sequencing. The strand marked with dUTP (the second cDNA strand) is not amplified, allowing strand-specific sequencing.

For high-throughput sequencing, the libraries were prepared following the manufacturer’s instructions and applied to an Illumina NovaSeq 6000 system for 150-nt paired-end sequencing.

### 2.7 RNA-seq raw data cleaning and alignment

First, raw reads containing more than 2-N bases were discarded. Then, adapters and low-quality bases were trimmed from raw sequencing reads using FASTX-Toolkit (Version 0.0.13). The short reads less than 16 nt were dropped as well. Afterward, clean reads were aligned to the GRCh38 genome by HISAT2 (Kim et al., 2015), allowing four mismatches. Uniquely mapped reads were used for gene read number counting and FPKM calculation (fragments per kilobase of transcript per million fragments mapped) (Trapnell et al., 2010).

### 2.8 Differentially expressed genes (DEG) analysis

The R Bioconductor package DESeq2 was applied to screen out the differentially expressed genes (DEGs) (Love et al., 2014). The P-value for correction <0.05 and fold change ≥2 or ≤0.5 were set as the cut-off criteria for identifying DEGs.

### 2.9 Batch effect correction and quality control

To minimize potential batch effects and technical variability in RNA-seq data, we applied ComBat_seq, an empirical Bayes method implemented in the “suva” R package, to adjust for known batch information across samples while preserving biological variance. Prior to batch correction, principal component analysis (PCA) was performed to visualize sample clustering and assess batch-related variation. After correction, PCA and hierarchical clustering confirmed improved consistency within experimental groups.

### 2.10 Alternative splicing analysis

The AS events and regulated alternative splicing events (RAS) between OE-*hnRNP-F* and NC samples were defined and quantified by using the splice sites usage variation analysis (SUVA) pipeline as described previously. Differential splicing of each pair of cells was analyzed. The frequency and reads proportion of the SUVA AS event (pSAR) of each AS event were calculated. For alternative splicing validation, we performed *RT-qPCR* on independent samples (*n* = 3 biological replicates) to confirm SUVA predictions, reporting both p-values and AS ratios in supplementary GraphPad data.

### 2.11 Functional enrichment analysis

In order to sort out functional categories of DEGs, Gene Ontology (GO) terms and KEGG pathways were identified using the KOBAS 2.0 server (Xie et al., 2011). The hypergeometric test and the Benjamini–Hochberg FDR controlling procedure were used to define the enrichment of each term.

### 2.12 Gene set enrichment analysis (GSEA)

GSEA is an analytical method for genome-wide expression profile microarray data. By comparing genes with predefined gene sets, it can identify functional enrichment. A gene set means a group of genes sharing localization, pathways, functions, or other features. GSEA was conducted using the clusterProfiler package (version 4.6.2). The fold change of gene expression between the Mets group and the Primary group was calculated, and the gene list was generated in accordance with the change of |log2FC|. Afterward, we utilized GSEA-based enriched *HALLMARK* gene sets of the Molecular Signature Database.

### 2.13 CLIP-seq data analysis

Public sequence data files of CLIP-seq data of *hnRNP-F* in human 293T cells from GSE34993 were downloaded from the Sequence Read Archive (SRA). After reads were aligned onto the genome, only uniquely mapped reads were used for the following analysis. The “ABLIRC” strategy was used to identify the binding regions of RBP on the genome (Xia et al., 2017). Reads with at least 1-bp overlap were clustered as peaks. For each gene, computational simulation was used to randomly generate reads with the same number and lengths as reads in peaks. The output reads were further mapped to the same genes to generate random max peak heights from overlapping reads. The whole process was repeated 500 times. All the observed peaks with heights higher than those of random max peaks (*P < 0.05) were selected. The target genes of *hnRNP-F* were finally determined by the peaks, and the binding motifs were called by HOMER software (Heinz et al., 2010).

### 2.14 Other statistical analyses

Principal component analysis (PCA) was performed by the R package factoextra (https://cloud.r-project.org/package=factoextra) to show the clustering of samples with the first two components. After controlling the reads by tags per million TPM) of each gene in samples, an in-house script (sogen) was used for visualization of next-generation sequence data and genomic annotations. The pheatmap package (https://cran.r-project.org/web/packages/pheatmap/index.html) in R was used to perform the clustering based on Euclidean distance.

### 2.15 Animal experiments

Seven-week-old male *db/db* mice (C57BLKS/J background, 12 weeks old, mean body weight: 45.2 ± 3.1 g) and their *db/m* littermates were purchased from GemPharmatech Co., Ltd. (Chengdu, China) and maintained in the specific pathogen-free (SPF) animal facility at Hubei University of Chinese Medicine (Wuhan, China). All experimental procedures involving animals were performed in strict compliance with the institutional guidelines and approved by the Animal Ethics Committee of Hubei University of Chinese Medicine (Approval No. HUCMS00303837). DKD modeling success was defined by (1) fasting blood glucose ≥16.7 mmol/L for three consecutive tests, (2) urine output >150% of controls, and (3) persistent proteinuria (Wang et al., 2021).

### 2.16 Statistical analysis

All results are presented as the average value plus or minus the standard deviation (SD). Statistical analyses were performed using GraphPad Prism 10.1.2 software (GraphPad, San Diego, CA). Differences between experimental groups were evaluated using either a paired two-tailed Student’s t-test or one-way ANOVA followed by Bonferroni’s *post hoc* test for multiple comparisons. A P-value of ≤0.05 was considered statistically significant.

## 3 Results

### 3.1 Effect of high-glucose on *hnRNP-F* protein level in HK2 cells

**FIGURE 1:**
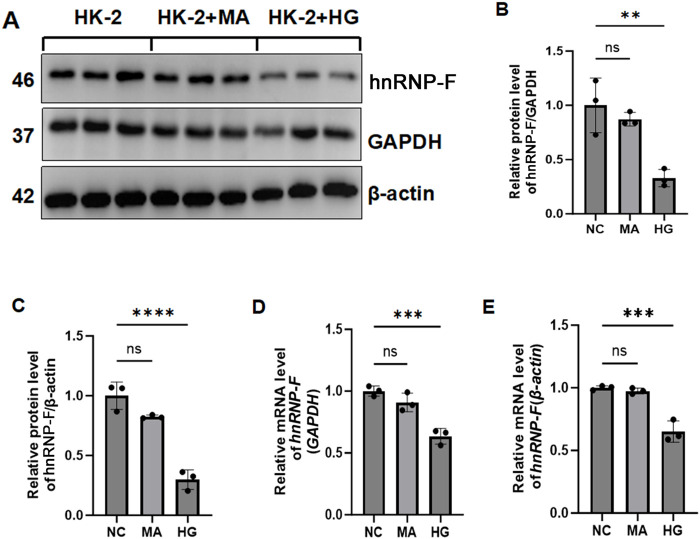
*hnRNP-F* expression in HK-2 cells under HG and hyperosmotic conditions. **(A)** Representative immunoblots of hnRNP-F protein in Con, Normal glucose (5.5 mM); HG, High-glucose (30 mM); Man, Hyperosmotic control (mannitol, 30 mM). **(B,C)** Quantitative analysis of hnRNP-F protein normalized to GAPDH or β-actin. **(D,E)** *hnRNP-F* mRNA levels normalized to *GAPDH* or *β-actin* by *RT-qPCR*. Error bars represent mean ± SEM. Statistical comparisons were performed using one-way ANOVA with *post hoc* tests; **P < 0.01, ***P < 0.001, ****P < 0.0001; ns: not significant.

In cells treated under normal glucose and hypertonic conditions, there was no significant difference in *hnRNP-F* protein levels between the two groups (*P* > 0.05). However, in HK-2 cells cultured with HG concentrations, *hnRNP-F* levels were significantly reduced (**P < 0.01) (Figures 1A–C). Western blot analysis demonstrated that high-glucose downregulated *hnRNP-F* expression. Mannitol, used as an osmotic control, exhibited no significant effect on *hnRNP-F* gene expression. Consistent with protein-level observations, *RT-qPCR* confirmed significant upregulation of *hnRNP-F* mRNA in high-glucose-treated HK-2 cells (*P < 0.05 vs. control) (Figures 1D,E), while treatment with equiosmolar mannitol showed no comparable effect (*P* > 0.05).

### 3.2 Overexpression of *hnRNP-F* regulates gene expression in high-glucose-treated renal tubular epithelial cells

**FIGURE 2:**
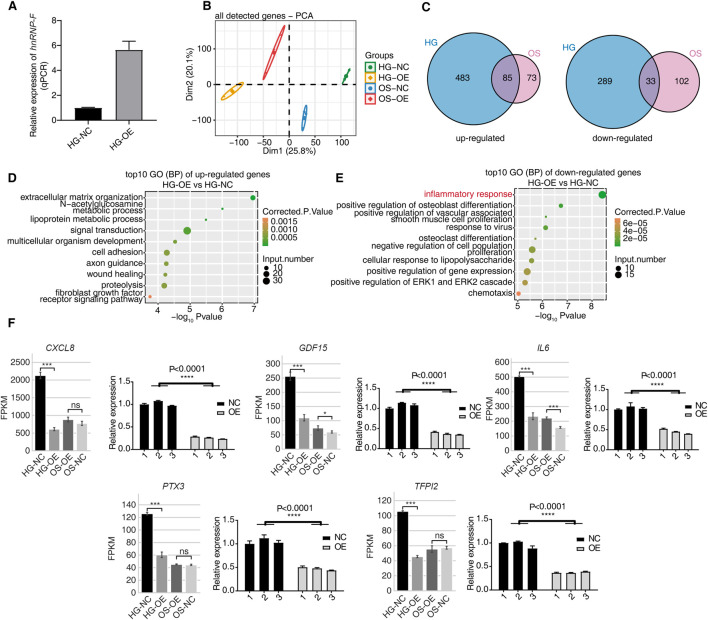
*hnRNP-F* regulates gene expression in HK-2 cells. **(A)** Verification of *hnRNP-F* mRNA expression in OE-*hnRNP-F* and NC HK2 cells under high-glucose conditions (HG-NC vs. HG-OE) using *RT-qPCR*. **(B)** Principal component analysis (PCA) of all samples based on normalized gene expression levels, showing clustering of HG-NC, HG-OE, OS-NC, and OS-OE groups. **(C)** Venn diagrams showing the overlap of differentially expressed genes (DEGs) between high-glucose (HG) and osmolality-control (OS) conditions. Left: upregulated genes; right: downregulated genes. **(D)** Dot plot displaying the top 10 enriched Gene Ontology (GO) biological process (BP) terms among upregulated genes in HG-OE vs. HG-NC. **(E)** Dot plot displaying the top 10 enriched GO biological process (BP) terms among downregulated genes in HG-OE vs. HG-NC. **(F)** Expression profiles of selected inflammation-related DEGs (*CXCL8, GDF15, IL6, PTX3*, and *TFPI2*) across four groups, shown as FPKM values from RNA-seq (left) and validated by *RT-qPCR* (right). Error bars represent mean ± SEM. Statistical comparisons were performed using one-way ANOVA with *post hoc* tests; *P < 0.05, **P < 0.01, ***P < 0.001, and ****P < 0.0001; ns, not significant.

Overexpression of *hnRNP-F* was constructed in HK2 cells cultured in a HG environment. qPCR results showed that *hnRNP-F* gene expression levels were upregulated in HK-2 cells after infection with overexpression of the *hnRNP-F* lentivirus (Figure 2A). To comprehensively investigate the *hnRNP-F*-mediated transcriptional regulation in high-glucose (HG) conditions, we constructed cDNA libraries prepared from control and *hnRNP-F*-overexpression cells (three biological replicates), which were incubated in high-glucose and mannitol. After removing adapters and contaminating sequences, we obtained a total of 742.7 million high-quality reads from each sample (Supplementary Table S2). Approximately 91.2%–96.39% paired-end reads per sample were then aligned to the human GRCH38 genome. RNA-seq yielded robust expression for 18,051 genes (Supplementary Table S3). PCA was carried out and revealed excellent clustering of expression gene changes between the OE-*hnRNP-F* HK-2 cells and control samples for different treatment conditions (Figure 2B). To evaluate the dynamics of gene expression between OE-*hnRNP-F* vs. Ctrl cells, we compared expression among all pairwise combinations of the samples using *DESeq2* (Love et al., 2014).

OE-*hnRNP-F* affects many gene expressions under HG conditions. A total of 890 DEGs were obtained (*P*-value of <0.05, fold change ≥2, or ≤0.5, FDR ≤0.05), of which 568 genes were upregulated and 322 genes were downregulated (Supplementary Table S4). Mannitol treatment was found to affect the expression of some genes (Supplementary Table S5), while the number of DEGs was larger in HK-2 cells treated with high-glucose than in HK-2 cells treated with mannitol. A Venn diagram illustrating the profiles of DEGs reveals an overlap between glucose and mannitol treatment (Figure 2C). This analysis displays unique and overlapping sets of DEGs in *hnRNP-F* overexpressing cells under HG treatment. Venn diagram analysis revealed an intersection of 118 genes between the high-glucose-treated and mannitol-treated OE-*hnRNP-F* HK-2 cells. Mannitol did not drastically affect overall gene expression when used as an osmolar control treatment.

To correlate the *hnRNP-F*-regulated gene expression and biological functions under high-glucose, we subjected all 890 DEGs to GO annotation (Supplementary Tables S6, S7). In the biological processes (BPs) of GO analysis, the upregulated genes in the *OE-hnRNP-F* samples were highly enriched in the “extracellular matrix organization” and “cell adhesion” processes (Figure 2D). The downregulated genes were mainly enriched in the inflammatory response and other related biological processes, as well as “regulation of insulin secretion,” “response to ischemia,” “regulation of insulin secretion,” positive regulation of angiogenesis,” and “response to hypoxia,” which are closely related to the pathogenesis of DKD (Figure 2E; Supplementary Table S7). Among these, the decreased expression of inflammation-related genes was of particular interest. Representative genes from inflammatory-related genes (*CXCL8*, *IL6*, *GDF15*, *PTX3*, and *TFPI2*) were selected for *RT-qPCR* validation of their mRNA levels. *CXCL8* and *IL6* were found to be enriched in the tumor necrosis factor pathway (Supplementary Table S6)*.* KEGG pathway enrichment analysis was also performed (Supplementary Figures S1A,B; Supplementary Table S8). The downregulated genes were also enriched in the *TNF* signaling pathway (Supplementary Figure S1B). The GO enrichment pathway of differentially expressed genes following mannitol treatment in HK-2 cells with *hnRNP-F* overexpression differs from that observed under HG conditions (Supplementary Figures S1C,D). Compared with HK-2 cells treated with HG and transfected with the empty vector (HG-NC group), overexpression of *hnRNP-F* could significantly downregulate the expression of *CXCL8*, *IL6*, *GDF15*, *PTX3*, and *TFPI2* (****P < 0.0001). The qPCR results were consistent with RNA sequencing data (Figure 2F). The primers are listed in Supplementary Table S1. In the hyperosmotic mannitol control condition, the overexpression of *hnRNP-F* resulted in the significant downregulation of only two genes, *IL6* and *GDF15.* This suggests that the upregulation of *hnRNP-F* under normoglycemic conditions did not exert any significant effect. However, under hyperglycemic conditions, it led to a marked reduction in the expression of genes associated with the inflammatory response, particularly those involved in the TNF signaling pathway and the pathogenesis of DKD.

### 3.3 Overexpression of *hnRNP-F* downregulates the transcription of genes involved in the *TNF* signaling pathway under HG conditions

**FIGURE 3:**
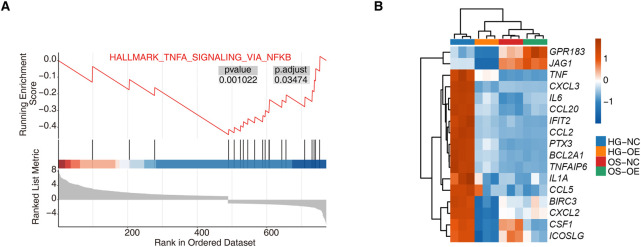
GSEA analysis for *hnRNP-F* overexpression in HK-2 cells. **(A)** Enrichment plot from gene set enrichment analysis (GSEA) showing significant negative enrichment of TNFα signaling via the NFκB pathway (HALLMARK_TNFα_SIGNALING_VIA_NFκB) in the HG-OE vs. the HG-NC group. **(B)** Hierarchical clustering heatmap showing the expression levels of TNFα/NFκB pathway-related genes across four sample groups: HG-NC, HG-OE, OS-NC, and OS-OE. Red and blue represent relative up- and downregulation, respectively.

We performed GSEA of genes differentially expressed upon *hnRNP-F* overexpression under high-glucose. The overexpression of *hnRNP-F* resulted in a significant inhibition of the *TNFα/NFκB* signaling pathway (Figure 3A). As demonstrated in Figure 3B, the upregulation of *hnRNP-F* resulted in a notable decrease in the expression of *PTX3* and *IL6*, both of which are genes associated with the *TNFα/NFκB* signaling pathway. Even in the hypertonic control of mannitol, overexpression of *hnRNP-F* inhibited the expression of these genes. The findings indicated that the upregulation of *hnRNP-F* suppressed the transcription of genes associated with the *TNF* signaling pathway, such as *CXCL8, IL6*, and *PTX3*.

### 3.4 Identification that *hnRNP-F* regulates alternative splicing events in HK-2 cells under high-glucose conditions

**FIGURE 4:**
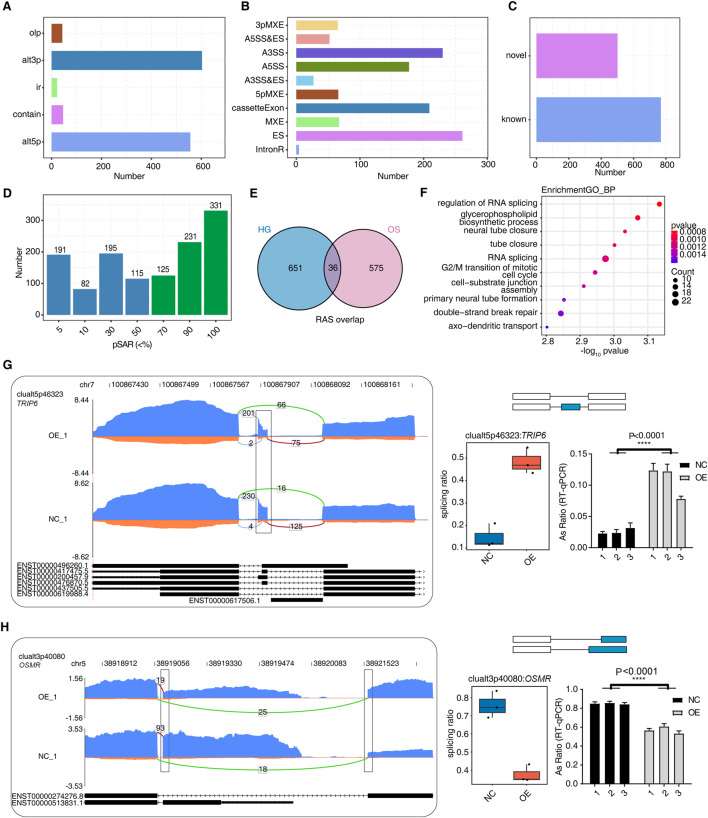
*hnRNP-F* regulates alternative splicing events in HK-2 cells. **(A)** Bar plot showing the number of regulated alternative splicing (RAS) events among OE and NC samples detected by SUVA. **(B)** Bar plot categorizing splice junctions constituting RAS events detected by SUVA into classical alternative splicing (AS) event types and displaying the number of each type. **(C)** Bar plot showing the number of known and novel RAS events. **(D)** Bar plot showing the number of RAS events with different abundances (pSAR) among all RAS with a frequency ≥50%. RAS with pSAR ≥50% are further analyzed and marked in red. **(E)** Venn diagram showing the overlap of high-confidence RAS events (pSAR ≥ 50%) identified under high-glucose (HG) and osmolality-control (OS) conditions. **(F)** The top 10 most enriched Gene Ontology (GO) terms related to biological processes are visualized for genes involved in RAS when comparing OE and NC samples. **(G)** Visualization of the read distribution of *TRIP6* in AS event clualt5p46323 from different groups, with splice junctions (SJs) labeled with SJ read numbers and the altered exon marked with a box. RNA-seq and *RT-qPCR* validation of the splicing ratio profile of the splicing event shown on the right. ****P < 0.0001. **(H)** Visualization of the read distribution of OSMR in AS event clualt3p40080 from different groups, with splice junctions labeled with SJ read numbers and altered splice sites marked with a box. RNA-seq and *RT-qPCR* validation of the splicing ratio profile of the splicing event shown on the right. ****P < 0.0001.

Given the multi-functional nature of *hnRNP-F* as an RNA-binding protein, our analysis also encompasses the impact of *hnRNP-F* on the regulation of AS events. We obtained a total of 125 million uniquely mapped reads from each sample, in which 33.15%–44.04% were junction reads (Supplementary Table S2). We analyzed the RNA-seq data using the software SUVA (9). Our analysis revealed the presence of distinct alternative splicing events (ASEs) and regulated alternative splicing events (RASEs) among the OE-*hnRNP-F* and control cells in the HG and mannitol-treated groups. Specifically, alt3p and alt5p were the main ASEs and RASEs between *OE-hnRNP-F* and control cells (Figure 4A; Supplementary Figure S3A; Supplementary Table S9. The SUVA-identified ASEs corresponded to the classical ASEs. *hnRNP-F* overexpression processing in HG cultured HK-2 cells resulted in a large number of differential variable splicing events, with a total of 1,158 significant RASEs detected. The main variable splicing event types included 65 3pMXE, 66 5pMXE, 230 A3SS, 27 A3SS&ES, 177 A5SS, 52 A5SS&ES, 261 ES, 4 IntronR, 67 MXE, and 209 cassette exons (Figure 4B; Supplementary Table S9). Mannitol treatment mainly affects the AS events of A3SS and ES (Supplementary Figure S3B; Supplementary Table S9). Figure 3C illustrates the presence of novel splicing events among the RASEs under HG conditions, a finding that aligns with the results observed following mannitol treatment (Supplementary Figure S3C). These findings suggest that the overexpression of *hnRNP-F* can modulate intracellular alternative splicing in response to hypertonic treatment conditions.

Due to a splicing event involving two transcripts, which may account for a very small proportion of the entire gene expression, our study focused on identifying the more dominant transcripts in splicing events. We specifically quantified the number of splicing events with varying proportions of RASEs in the region covered by all reads. We also excluded splicing events with a low proportion (pSAR<50%). A total of 687 events with pSAR>50% were detected in overexpression *hnRNP-F* cells treated with high-glucose (Figure 4D). A total of 611 events were detected in the cells treated with mannitol (Supplementary Figure S3D). While a diversity of AS events was observed in cells treated with mannitol, the functional disparities in these splicing events between the two groups were pronounced when compared to the HG group. Notably, only 36 AS events were common to both treatment conditions (Figure 4E). This result indicated that overexpressed *hnRNP-F* HK-2 cells have a distinct AS profile in response to HG exposure. Gene Ontology biological process (GO-BP) enrichment analysis revealed that the HG group exhibited significant alterations in alternative splicing, predominantly enriched in pathways related to “regulation of RNA splicing” and “RNA splicing” (Figure 4F). The AS events of the mannitol group were mainly enriched in the “microvillus assembly” and “epithelial tube formation” pathway (Supplementary Figure S3E). To validate the accuracy of the predicted *hnRNP-F*-regulated ASEs selected from the RNA-seq data under the HG condition, two RASEs were selected for verification. The ratio of variable splicing events occurring in the gene *OSMR* (alt3p) decreased in the OE-*HNRNP-F* group (Figure 4H), and increased in the gene *TRIP6* (alt5p) (Figure 4G), as expected. We present the designed PCR primer pairs in Supplementary Table S1. *TRIP6* mediates inflammatory response and renal fibrosis in diabetic nephropathy (Lin et al., 2021).

### 3.5 *hnRNP-F* CLIP-seq reads revealed that *hnRNP-F* bound to splicing factors and regulated alternative splicing events

**FIGURE 5:**
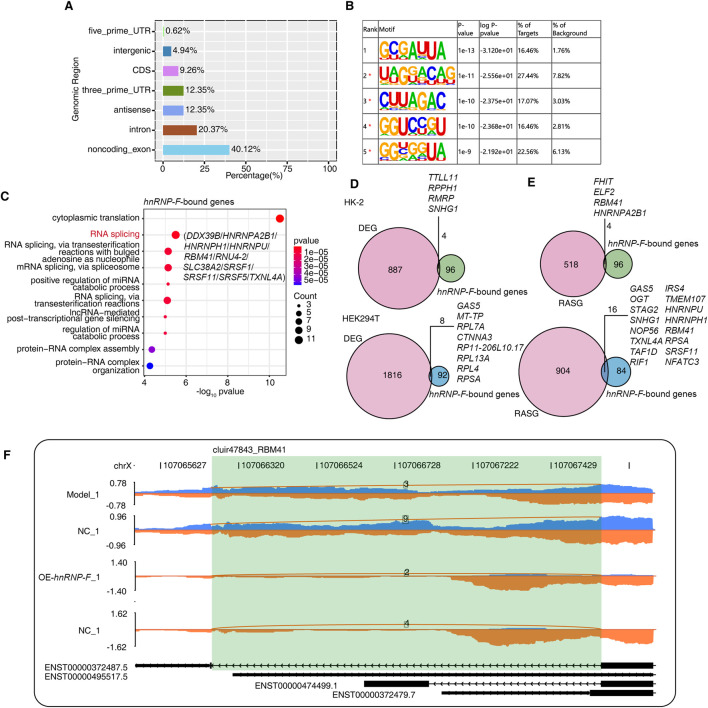
*hnRNP-F* binds to other splicing factors and regulates alternative splicing of *RBM41*. **(A)** Bar plot showing the distribution of genomic regions of *HNRNP-F*-bound peaks. **(B)** The five most enriched motif sequences among the *HNRNP-F*-bound peaks were identified using HOMER software. **(C)** Dot plot showing the top 10 enriched GO biological processes of genes bound by *HNRNP-F*. **(D)** Venn diagram illustrating the overlap between *HNRNP-F*-bound genes and *HNRNP-F*-regulated differentially expressed genes (DEGs) in HK-2 cells and 293T cells (Up HK-2 cells; Down 293T cells). **(E)** Venn diagram illustrating the overlap between *HNRNP-F*-bound genes and *HNRNP-F*-regulated alternatively spliced genes in HK-2 cells and 293T cells (Up HK-2 cells; Down 293T cells). **(F)** Visualization of the read distribution of *RBM41* in the AS event cluir47843 from different groups, with splice junctions labeled with the SJ read number. The model and NC were derived from the RNA-seq data of 293T cells, while OE-*HNRNP-F* and NC were derived from the RNA-seq data of HK-2 cells.

The *hnRNP-F* CLIP-seq data in human 293T cells were obtained from the SRA database accession number GSE34993. These data were utilized to identify transcripts that interact with *hnRNP-F* in cells. Only reads that mapped uniquely were included in the subsequent analysis. Comparisons between the control group and the IP groups revealed that the reads in the latter were predominantly enriched in noncoding exons, introns, and the 3′UTR region (Figure 5A). RNA-binding proteins that bind to the 3′UTR region often have an impact on RNA stability, suggesting that *hnRNP-F* may influence RNA stability.

Hypergeometric Optimization of Motif EnRichment (HOMER v4.11, http://homer.ucsd.edu/homer/) was employed for motif analysis of the specific binding peaks identified in the experimental samples. The motif enrichment analysis of the immunoprecipitation (IP) groups revealed enrichment of the UA-rich motif 5′-UUA-3′ in the *hnRNP-F-*bound motif (Figure 5B). Subsequently, the gene sequences corresponding to the bound peak clusters were aligned with the GO database for annotation, which indicated enrichment ion the RNA splicing process. These primarily included the heterogeneous ribonucleoprotein (*HNRNP*) family as well as *SRSF* splicing factors, including *HNRNPA2B1*, *HNRNPH, HNRNPU, SRSF1, SRSF5,* and *SRSF11* (Figure 5C). Studies have shown that *HNRNPA2B1*-binding motifs were UA rich (Wu et al., 2018).

Next, we asked whether differences in *hnRNP-F* binding genes were associated with different gene expressions. We performed gene-based differential binding analyses. We separately analyzed the transcriptome data of *hnRNP-F* in human 293T cells (GSE34995) and the transcriptome data of *hnRNP-F* in HK-2 cells that we independently measured. The results showed that *hnRNP-F*-binding genes overlap with differently expressed genes. Of particular interest was the observation that the lncRNA *SNHG1*, when bound by *hnRNP-F* in 293T cells, exhibited differential expression in HK-2 cells overexpressing *hnRNP-F* (OE-hnRNP-F). Otherwise, in 293T cells, the lncRNA *SNHG1* underwent alternative splicing (Figures 5D,E). We also performed an association analysis utilizing CLIP-seq and AS methodologies, which revealed that AS events occurred in four gene regions where *hnRNP-F* binds in HK-2 and in 16 gene regions in 293T cells (Figure 5E). The AS events of gene *RBM41* were detected in both cells (Figure 5E). The analysis of distribution maps indicated that the overexpression of *hnRNP-F* in HK-2 cells led to a diverse intron retention (ir) AS event of *RBM41*. Similarly, in 293T cells with *hnRNP-F* knockdown, an ir AS event was also observed in *RBM41*. There is an *hnRNP-F* bound site near the splicing site (Figure 5F). The findings suggest that *hnRNP-F* can interact with *RBM41*. The interaction between *hnRNP-F* and *RBM41* results in the production of a truncated transcript of *RBM41*. Our hypothesis posits that the truncated transcript generated by *RBM41* could potentially influence the AS events. Nonetheless, given that the experiment conducted in 293T cells involved the knockdown of *hnRNP-F* and was characterized by a relatively low sequencing depth, this AS event warrants further experimental investigation.

### 3.6 Experimental validation of *hnRNP-F*-regulated differential gene expression in a *db/db* mouse model

**FIGURE 6:**
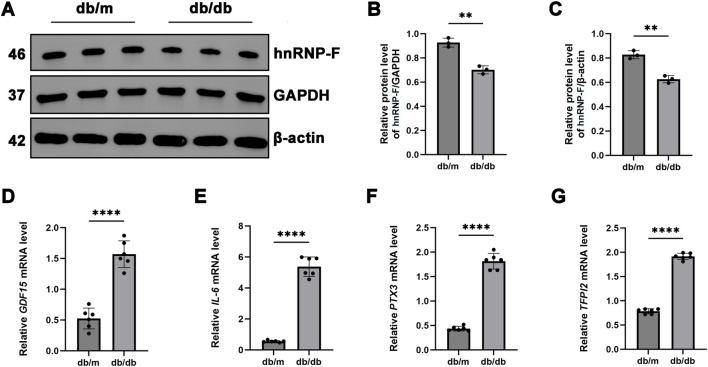
Expression of *hnRNP-F* in the kidneys of *db/db* mice and validation of its differentially expressed genes. **(A)** Representative immunoblot images showing hnRNP-F protein expression in the kidneys of *db/db* and *db/m* mice. **(B,C)** Quantification of hnRNP-F protein levels normalized to GAPDH or β-actin as internal controls. **(D–G)** mRNA levels of *hnRNP-F*-modulated differentially expressed genes quantified by *RT-qPCR*. Error bars represent mean ± SEM. Statistical comparisons were performed using one-way ANOVA with *post hoc* tests; **P < 0.01, ****P < 0.0001; ns: not significant.

Initially, we observed a significant reduction in the levels of *hnRNP-F* protein in the kidney of *db/db* mice compared to *db/m* controls (***P < 0.001) (Figures 6A–C). Subsequently, we validated the differentially expressed genes identified through RNA-seq (DEGs: *CXCL8, GDF15, IL6, PTX3,* and *TFPI2*) by *RT-qPCR*. Notably, *CXCL8* is a chemokine specific to humans and lacks a direct ortholog in mice, which precludes its validation in murine models. Compared to *db/m* controls, *db/db* mice demonstrated significantly elevated renal expression of genes associated with inflammation (*GDF15*, *IL6*, *PTX3*, and *TFPI2*, ****P < 0.0001) (Figures 6D–G), suggesting their critical roles in the progression of DKD. The downregulation of *hnRNP-F* protein may have facilitated the upregulation of these genes in the *db/db* mouse model.

### 3.7 *hnRNP-F* overexpression exerts anti-inflammatory effects in MPC5 cells under HG conditions

**FIGURE 7:**
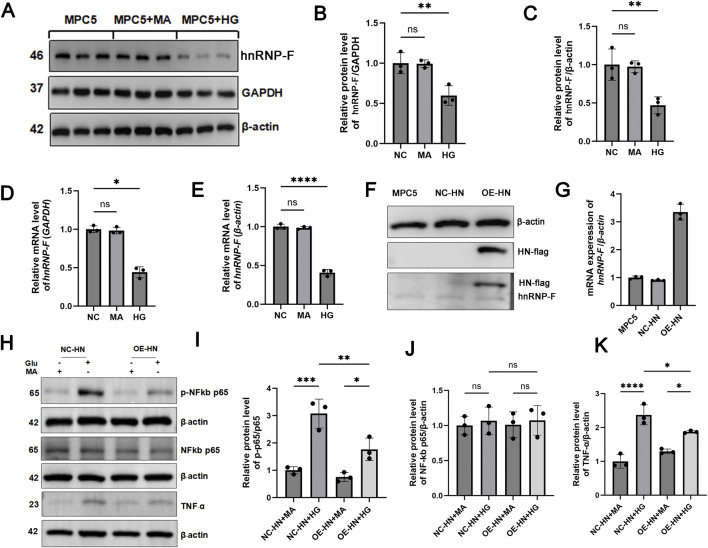
Role of *hnRNP-F* in MPC-5 cells under high-glucose and its regulation of *TNF-α/NF-κB* signaling. **(A)** Representative Western blot analysis of hnRNP-F protein in NG: Normal glucose (5.5 mM), HG: High-glucose (30 mM), Man: Hyperosmotic control (mannitol, 30 mM). **(B,C)** Quantitative analysis of hnRNP-F protein normalized to GAPDH or β-actin. **(D,E)** *hnRNP-F* mRNA levels normalized to *GAPDH* or *β-actin* by *RT-qPCR*. **(F)** *hnRNP-F* protein in vector-transfected (NC) and *hnRNP-F-*overexpressing (OE) MPC-5 cells under high-glucose. **(G)** *hnRNP-F* mRNA levels in NC and OE cells under high-glucose. **(H)** Western blot analysis of TNF-α/NF-κB pathway components in NC and OE cells: TNF-α, Total p65, Phospho-p65 (Ser536). **(I)** Quantitative analysis of Phospho-p65/total p65 ratio. **(J)** Quantitative analysis of p65/β-actin. **(K)** Quantitative analysis of TNF-α/β-actin. Error bars represent mean ± SEM. Statistical comparisons were performed using one-way ANOVA with *post hoc* tests; *P < 0.05, **P < 0.01, ***P < 0.001, and ****P < 0.0001; ns, not significant.

Under HG conditions, MPC-5 cells demonstrated a significant reduction in *hnRNP-F* protein levels (**P < 0.01), with minimal correlation to hyperosmolarity induced by mannitol (Figures 7A–E). In cells stably transfected to overexpress *hnRNP-F* (validated by immunoblotting) (Figures 7F,G), *TNF-α* expression was significantly attenuated in HG conditions (*P < 0.05) (Figures 7H,K), and *NF-κB p-p65* phosphorylation was notably suppressed (**P < 0.01) (Figures 7H–J).

### 3.8 Effect of LPS on *hnRNP-F* expression and anti-inflammatory effects of *hnRNP-F* overexpression in LPS-treated MPC5 cells

**FIGURE 8:**
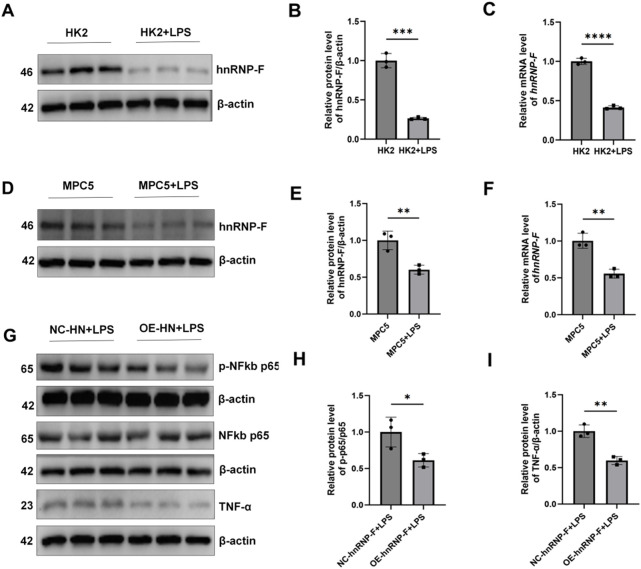
Expression of *hnRNP-F* in HK2 cells and MPC-5 cells under LPS conditions and its regulation of the TNF-α/NF-κB signaling pathway in MPC-5 cells. **(A)** Representative immunoblot images of hnRNP-F protein expression in HK-2 cells under LPS conditions (10 µg/mL) and normal conditions. **(B)** Quantitative analysis of hnRNP-F protein normalized to β-actin in HK-2 cells under normal and LPS conditions. **(C)** *hnRNP-F* mRNA levels in HK-2 cells under normal and LPS conditions were quantified by RT-qPCR and normalized to *β-actin*. **(D)** Representative immunoblot images of hnRNP-F protein expression in MPC5 cells under LPS conditions (45 µg/mL) and normal conditions. **(E)** Quantitative analysis of hnRNP-F protein normalized to β-actin in MPC5 cells under normal and LPS conditions. **(F)** *hnRNP-F* mRNA levels in MPC5 cells under normal and LPS conditions were quantified by RT-qPCR and normalized to *β-actin*. **(G)** Western blot of TNF-α/NF-κB pathway components in NC-hnRNP-F and OE-hnRNP-F MPC5 cells: TNF-α, total p65, phosphorylated p65 (Ser536). **(H)** Quantitative analysis of the phosphorylated p65/total p65 ratio in MPC5 cells under basal and LPS conditions. **(I)** Quantitative analysis of TNF-α/β-actin in MPC5 cells under basal and LPS conditions. Error bars represent mean ± SEM. Statistical comparisons were performed using one-way ANOVA with *post hoc* tests; *P < 0.05, **P < 0.01, ***P < 0.001, ****P < 0.0001; ns, not significant.

Under LPS-induced inflammatory conditions, *hnRNP-F* protein levels were significantly decreased in both HK-2 cells and MPC-5 podocytes (*P < 0.001; *P* < 0.01; Figures 8A–F). In MPC-5 cells stably transfected to overexpress *hnRNP-F*, TNF-α expression was markedly attenuated (*P < 0.05) (Figure 8I), and NF-κB p-p65/p65 was significantly suppressed (**P < 0.01) (Figures 8G,H) under LPS exposure.

### 3.9 *hnRNP-F* is physically associated with *ZFP36* to form a complex that regulates gene expression and alternative splicing

**FIGURE 9:**
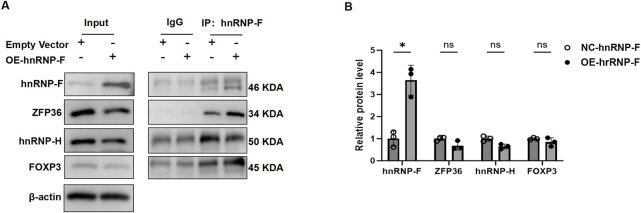
hnRNP-F associates with *ZFP36* to form a complex that modulates gene expression and splicing. **(A)** Co-IP assays in HK-2 cells transfected with empty vector (NC) or hnRNP-F overexpression plasmid (OE-hnRNP-F). Whole-cell lysates (Input) and immunoprecipitated complexes (IP: α-hnRNP-F) were probed for hnRNP-F (46-/50-kDa isoforms), ZFP36 (34 kDa), and FOXP3 (45 kDa). IgG served as a negative control, and β-actin (42 kDa) was the loading control. **(B)** Quantitative analysis of protein enrichment in Co-IP complexes. Relative protein levels were normalized to IgG control (mean ± SD; *n* = 3). **P < 0.01 vs. NC group; ns: not significant (*P* > 0.05), two-tailed Student’s t-test.

To elucidate the mechanistic role of *hnRNP-F* in transcriptional repression, we conducted Co-IP experiments to examine the *hnRNP-F* interactome *in vivo*. In these experiments, HK-2 cells were engineered to stably overexpress *hnRNP-F*. Total protein lysates were subjected to immunoprecipitation using antibodies specific to *hnRNP-F*, followed by WB with antibodies targeting *ZFP36, HNRNPH*, and *FOXP3* (Figure 9). The Co-IP analysis using *hnRNP-F*antibodies, followed by WB with *ZFP36* antibodies, demonstrated a physical association between *hnRNP-F* and *ZFP36.* The *ZFP36* gene, also known as tristetraprolin (*TTP*), is a crucial RNA-binding protein that plays a vital role in various biological processes. *ZFP36* modulates mRNA stability through its interaction with AU-rich elements (AREs) within mRNA, consequently affecting gene expression and cellular function (Makita et al., 2021). Furthermore, empirical evidence suggests that *ZFP36* plays a substantial role in the regulation of alternative splicing (Tu et al., 2019; Chan et al., 2025). We speculate that *hnRNP-F* and *ZFP36* form a complex that regulates gene expression and alternative splicing.

## 4 Discussion

The pathophysiology of DKD involves multiple pathways, such as hemodynamic, metabolic, and inflammatory pathways. Targeting inflammatory and fibrotic pathways may have important therapeutic implications in DKD research (Matoba et al., 2019). Therefore, it is necessary to further investigate its molecular regulatory mechanism in cells associated with DKD to provide new ideas for finding new therapeutic targets for DKD.

As an RNA-binding protein, *hnRNP-F* is capable of binding to mRNA and participating in the post-transcriptional regulation of target genes, and it is known to modulate the expression of target genes. Abnormal expression of *hnRNP-F* has been reported to have a significant effect on the progression of diabetic nephropathy, and high expression of *hnRNP-F* may have a better protective effect. In patients with type 2 diabetes, the protein levels of *hnRNP-F* have significantly decreased in renal cortex tissues, but the role of *hnRNP-F* in renal tubular epithelial cell mechanisms remains unclear (Liao et al., 2024). RNA-seq analysis in this study demonstrated that *hnRNP-F* broadly modulates high-glucose-induced differential gene expression and alternative splicing in HK-2 cells. Downregulation of *hnRNP-F* expression under HG conditions or in DKD was subsequently confirmed across HK-2 cells, MPC5 cells, and *db/db* mouse models. Furthermore, lentivirus-mediated *hnRNP-F* overexpression in MPC5 cells significantly suppressed the TNF-α/NF-κB signaling pathway. The expression and synthesis of *TNF-α*, a potent inflammatory factor, are not only limited to hematopoietic cells, but also can be produced by renal intrinsic cells, such as mesangial cells, endothelial cells, tubular epithelial cells, etc. (Jevnikar et al., 1991; Sugimoto et al., 1999). *TNF-α* plays an activating role in renal intrinsic cells, including a second messenger system, transcription factors, and cytokines, and participates in the synthesis of inflammatory mediators and tissue-compatible complexes (Vielhauer et al., 2005). It has been demonstrated that insulin-resistant diabetic patients have increased serum levels of *TNF-α* (Iwata et al., 2001), and the levels of *TNF-α* in the blood or glomerular cells are considered to correlate with the damage to the tethered cells in patients with DKD. Being a pleiotropic transcription factor, *NF-κB* is a regulatory hub for thylakoid cells to express a variety of immune-inflammation-related genes, and is intimately implicated in thylakoid cell proliferation and secretion of inflammatory factors (Evans et al., 2002). Numerous studies have shown that *NF-κB* may accelerate the progression of DKD by regulating inflammation; for example, it has been shown that *NF-κB* mediates high-glucose-induced inflammatory response and ECM accumulation in glomerular mesangial cells (Chen et al., 2016; Liu et al., 2019).

We considered that the significant downregulation of *CXCL8, IL6, GDF15*, *PTX3*, and *TFPI2* warranted additional focus. Existing studies have reported that inhibition of *CXCL8* attenuates high-glucose-induced renal tubular cell-mediated inflammation and apoptosis in diabetic kidney disease (Bai et al., 2022). Significantly, activation of *CXCL8* has been demonstrated to heighten TNF-α-induced inflammatory responses (Huang et al., 2018). *IL-6* signaling is known to be involved in the core inflammatory response in the progression of DKD (Feigerlová and Battaglia-Hsu, 2017). As is known, *TNF-α* inhibits the transcription factors resulting in the production of *IL-6* (Tanaka et al., 2014). Growth differentiation factor-15 (*GDF-15*) increases the likelihood of DKD by affecting reno-protective factors with anti-inflammatory activity (Delrue et al., 2023). GDF-15 inhibits inflammation by reducing the infiltration of inflammatory cells, diminishing the secretion of cytokines and chemokines, and attenuating macrophage and T cell activity to suppress the release of *TNF-α*, *IL-6*, and *IL-1β* (Tang et al., 2024). In addition, it has been shown that *TNF-α* could increase the transcriptional activity of *GDF-15* by potentiating multiple signal transduction pathways, especially the classical *NF-κB* and *MAPK* pathways (Adela et al., 2015).

Two other genes, *PTX3* and *TFPI2*, were also involved in the DKD pathological process. *PTX3* induces mitochondrial dysfunction and renal tubular cell senescence via β-linker activation, leading to renal fibrosis (Luo et al., 2023). It was reported that *TFPI2* can regulate the endothelial–mesenchymal transition and the *TGF-β2* signaling pathway and is a potential promoter of DKD pathogenesis (Guan et al., 2022). We also downloaded the CLIP-seq data of *hnRNP-F* and found that *hnRNP-F* and lncRNA *SNHG1* had the potential to combine. Meanwhile, the expression of lncRNA *SNHG1* was downregulated after *hnRNP-F* overexpression. *SNHG1* is an annotated lncRNA, which is mainly localized in the nucleus. It is well established that *SNHG1* interacts with the promoter regions of its downstream genes to enhance their expression (Li et al., 2020; Sun et al., 2017). The present study demonstrates that overexpression of *hnRNP-F* results in decreased levels of *SNHG1* expression. Additionally, the expression of certain TNFα-related genes is suppressed following *hnRNP-F* overexpression. These findings suggest a potential interaction between *hnRNP-F* and *SNHG1* in regulating the transcription of these target genes.

**FIGURE 10:**
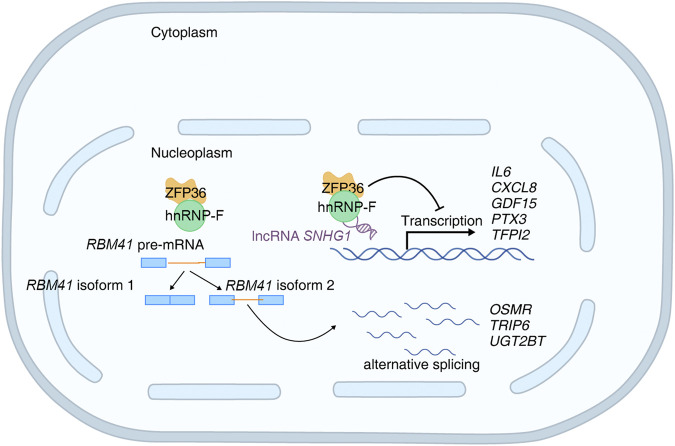
Schematic diagram of hnRNP-F-mediated regulation of gene expression and alternative splicing in high-glucose.

Our present experiments revealed that *hnRNP-F* combined with lncRNA *SNHG1* in high-glucose-induced renal tubular epithelial cells significantly reduced the expression of genes associated with the *TNFα/NFκB* signaling pathway or with DKD pathogenesis (Figure 3). The mechanism of this transcriptional repression requires further investigation. Undoubtedly, previous studies indicate that *hnRNP-F* interacts with multiple proteins, including *hnRNP-H*, *FOXP3*, and tristetraprolin (*TTP*, also known as *ZFP36*) (Reznik et al., 2014). Co-IP assays performed in HK2 cells confirmed a physical interaction between *hnRNP-F* and *ZFP36* (*P < 0.05 vs. IgG control). However, no interaction was detected between *hnRNP-F* and *FOXP3.* While *hnRNP-H* co-precipitated with *hnRNP-F*, its comigration with the antibody heavy chain (∼50 kDa) precluded definitive assessment of this interaction. Notably, overexpression of *hnRNP-F* did not significantly alter the protein levels of *ZFP36, FOXP3,* or *hnRNP-H*, as determined by densitometric analysis (Figure 10). A prior study suggested that *hnRNP-F* acts as a co-factor with *TTP* to increase ARE-mRNA decay. The current study hypothesizes that *hnRNP-F* and *TTP* form a complex mediated by *SNHG1* to regulate gene expression. Nevertheless, further experimental evidence is necessary to substantiate this conclusion.

*hnRNP-F*, as a coregulator of alternative splicing, always interacts with other RNA-binding proteins, including *RBM41*. We found that *hnRNP-F* overexpression notably promotes several alternative RNA-binding protein splicings. We observed that *hnRNP-F*-dependent alternative splicing of *RBM41* generates a short isoform in *hnRNP-F* overexpression cells. RBM41 is the paralog of U11/U12-65K, a known unique component of the U11/U12 di-snRNP. Both proteins utilize their highly similar C-terminal RRM domains to bind the 3′-terminal stem-loops in U12 and U6atac snRNAs with comparable affinity. Recent studies identify RBM41 as a novel, unique protein component of the minor spliceosome, functioning in post-splicing steps and the disassembly process of the minor spliceosome (Norppa et al., 2024; Taira et al., 2025). It is speculated that *hnRNP-F* may affect the alternative splicing of RBM41, thereby influencing the overall post-transcriptional regulatory pattern within the cell.

We also found that *hnRNP-F* overexpression significantly alters variable exons of *OSMR* and *UGT2B7*. *OSMR* is a receptor for *OSM,* and *OSM* signaling plays a role in fibrosis, including inflammation, vascular dysfunction, and fibroblast activation (Stawski and Trojanowska, 2019). Miroslav Dostalek et al. discovered that diabetes reduces *UGT2B7* enzymatic activity in the kidney (Dostalek et al., 2011).

*hnRNP-F* also affects the inclusion or deletion of exons in some genes, resulting in transcripts of different lengths, like gene *TRIP6* and *IRF3. TRIP6* mediates inflammatory response and fibrosis in diabetic nephropathy (Lin et al., 2021). As a key molecule in the interferon gene/interferon regulatory factor 3 (*STING/IRF3*) signaling pathway, *IRF3* is involved in mediating the inflammatory response at different stages of DKD progression (El-Deeb et al., 2023). Analysis of CLIP-seq data from *hnRNP-F* showed that *hnRNP-F* specifically binds to some *hnRNP* family proteins and splicing factors. Based on the above results, we speculated that *hnRNP-F* may mediate variable splicing in high-glucose-induced HK2 cells through interaction with *hnRNP* family proteins (Figure 5).

In summary, *hnRNP-F* could have dual functions in mRNA transcriptional and post-transcriptional levels. We find that *hnRNP-F* may bind with lncRNA *SNHG1* to negatively regulate the transcription of genes involved in the *TNFα/NFκB* signaling pathway. Interestingly, *hnRNP-F* also regulates the alternative splicing of *hnRNP* proteins and splicing factors. This finding suggests that *hnRNP-F* may play a role in DKD by regulating the differential expression and variable splicing of genes associated with diabetic nephropathy, especially genes associated with inflammatory response; however, its exact mechanism requires further experimental verification.

## Data Availability

The data presented in the study are deposited in the GEO repository, accession numbers GSE273001 and GSE299230.
