# Supplementary figures and images for: Integrative RNA-seq and CLIP-seq analysis reveals hnRNP-F regulation of TNFα/NFκB signaling in high-glucose conditions
Source: Front Physiol. 2025 Sep 9;16:1475441. doi: 10.3389/fphys.2025.1475441 (PMC12454069; doi:10.3389/fphys.2025.1475441)

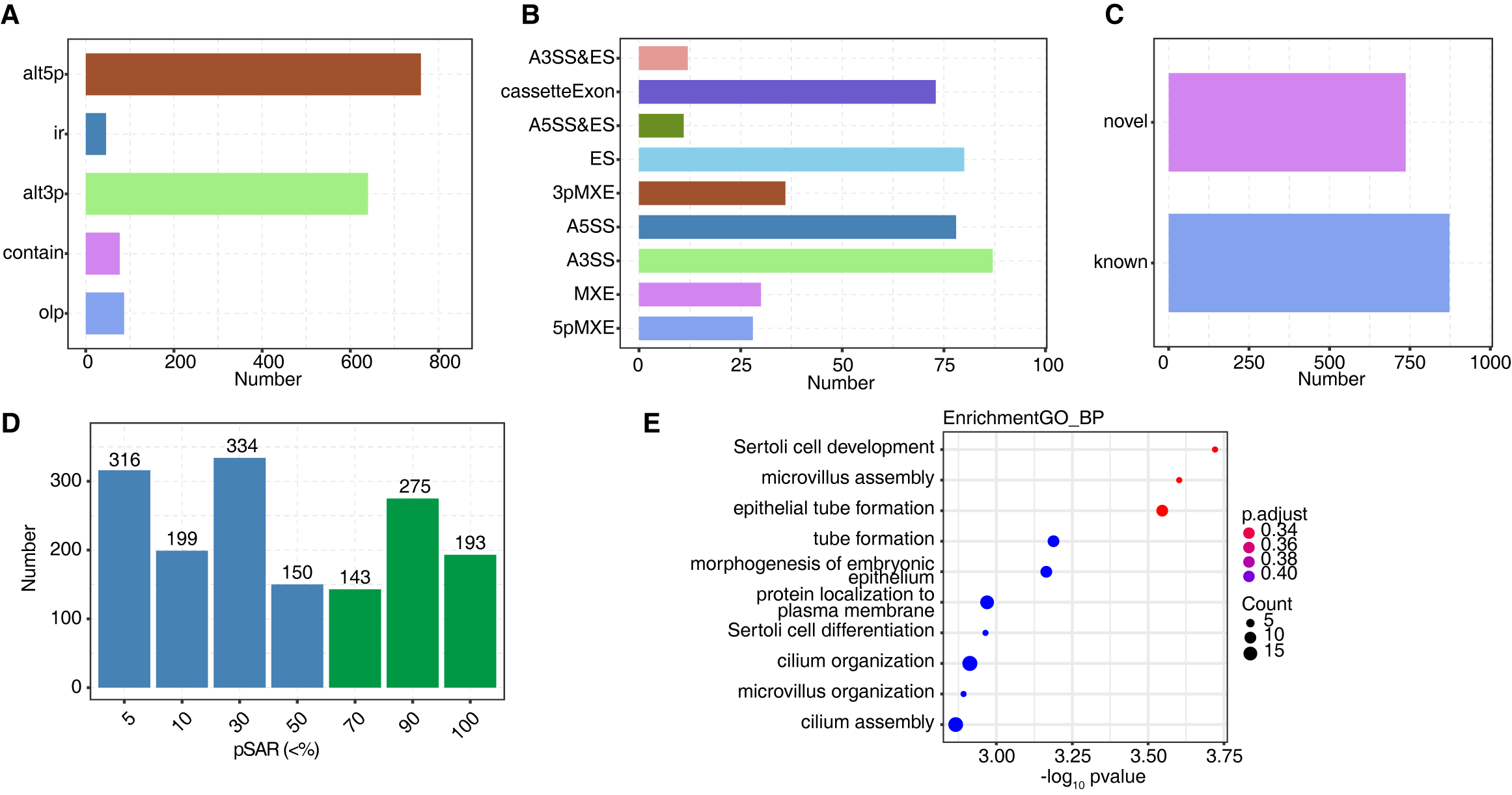

Supplement: Supplementary file 4 [file Image3.tif]

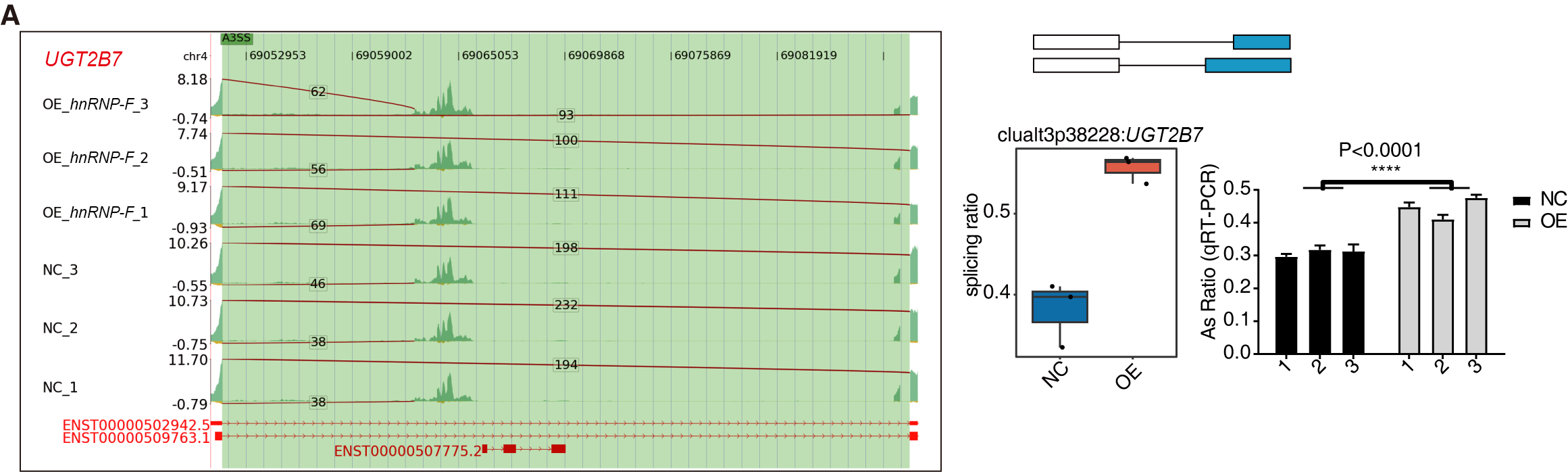

Supplement: Supplementary file 6 [file Image2.tif]

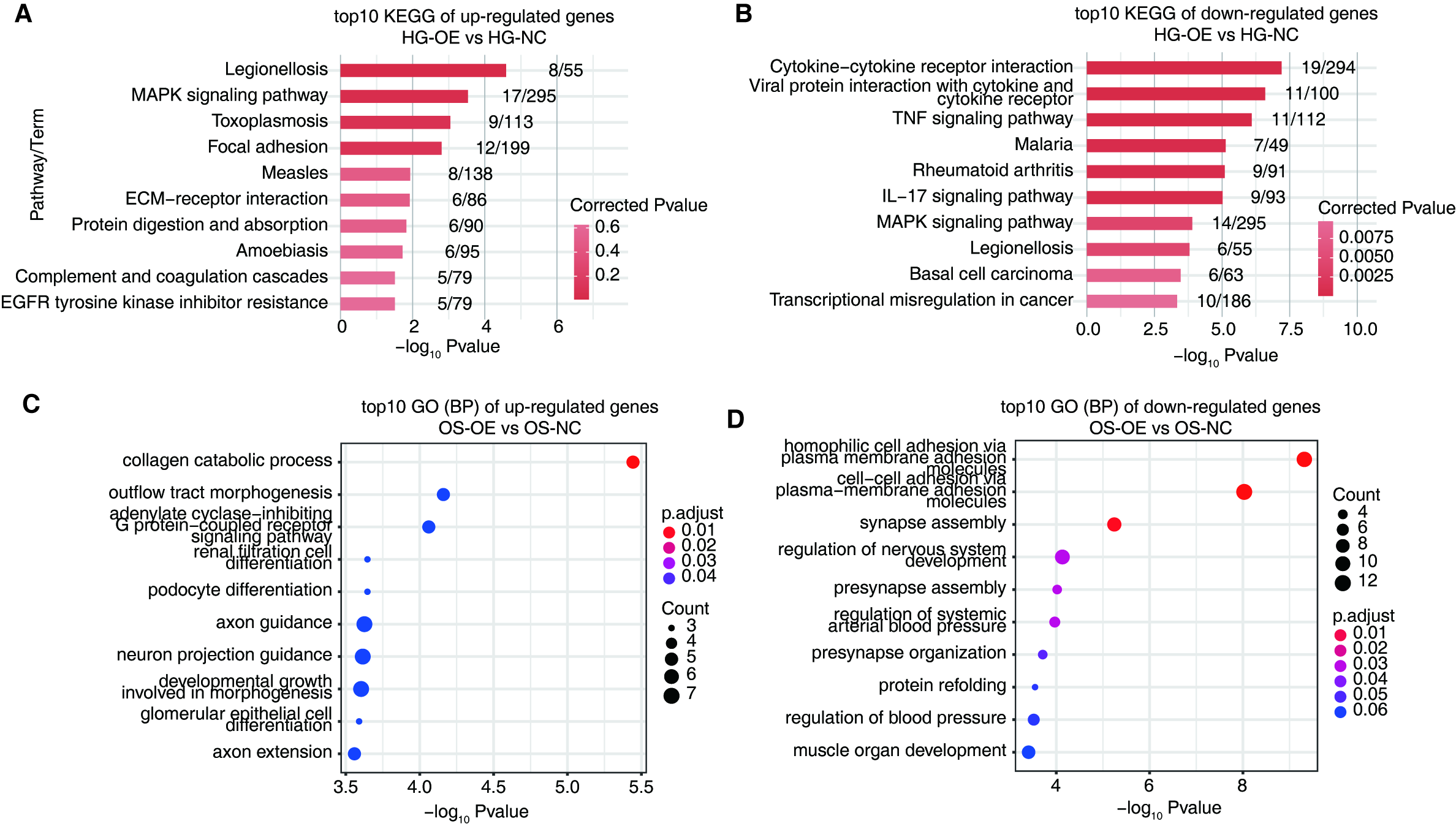

Supplement: Supplementary file 7 [file Image1.tif]
