# Supplementary material for: Integrative RNA-seq and CLIP-seq analysis reveals hnRNP-F regulation of TNFα/NFκB signaling in high-glucose conditions
Source: Front Physiol. 2025 Sep 9;16:1475441. doi: 10.3389/fphys.2025.1475441 (PMC12454069; doi:10.3389/fphys.2025.1475441)
Supplement: Supplementary file 10 [file Table2.docx]

Supplementary table 2: Summary of RNA-seq reads used in analysis

| Sample | HG-OE-hnRNPF_1st | HG-OE-hnRNPF_2nd | HG-OE-hnRNPF_3rd | HG-Ctrl_1st | HG-Ctrl_2nd | HG-Ctrl_3rd | OS-OE-hnRNPF_1st | OS-OE-hnRNPF_2nd | OS-OE-hnRNPF_3rd | OS-Ctrl_1st | OS-Ctrl_2nd | OS-Ctrl_3rd |
| --- | --- | --- | --- | --- | --- | --- | --- | --- | --- | --- | --- | --- |
| Clean reads | 69164318 | 70999146 | 76058654 | 60409234 | 64117866 | 65853146 | 56166342 | 57545708 | 55489076 | 51800640 | 54891556 | 60200020 |
| Total mapped | 67442970  (97.51%) | 69066910  (97.28%) | 71913474 (94.55%) | 58821346 (97.37%) | 62378988 (97.29%) | 64077158 (97.3%) | 52743270  (93.91%) | 53986764  (93.82%) | 51906424  (93.54%) | 48190326  (93.03%) | 50951294  (92.82%) | 55800028  (92.69%) |
| Total Uniquely mapped | 64614180 (95.81%) | 65702248 (95.13%) | 65642832 (91.28%) | 56624388 (96.27%) | 60127986 (96.39%) | 61765622 (96.39%) | 51278948  (97.22%) | 52599260  (97.43%) | 50322568  (96.95%) | 46823726  (97.16%) | 49760694  (97.66%) | 54239852  (97.2%) |
| Splice reads | 21747075 (33.66%) | 22095721 (33.63%) | 22149709 (33.74%) | 18853597 (33.3%) | 19933862 (33.15%) | 20587987 (33.33%) | 21530901  (41.99%) | 22234590  (42.27%) | 21540122  (42.8%) | 20089065  (42.9%) | 21913310  (44.04%) | 23372536  (43.09%) |
